# Virucidal Efficacy of Blue LED and Far-UVC Light Disinfection against Feline Infectious Peritonitis Virus as a Model for SARS-CoV-2

**DOI:** 10.3390/v13081436

**Published:** 2021-07-23

**Authors:** Amanda Gardner, Sayani Ghosh, Magdalena Dunowska, Gale Brightwell

**Affiliations:** 1 AgResearch Ltd., Hopkirk Research Institute, Massey University, Corner University Ave and Library Road, Palmerston North 4442, New Zealand; amanda.gardner@agresearch.co.nz; 2 School of Veterinary Science, Massey University Manawatu (Turitea), Tennent Drive, Palmerston North 4474, New Zealand; S.Ghosh1@massey.ac.nz (S.G.); M.Dunowska@massey.ac.nz (M.D.); 3 New Zealand Food Safety Science and Research Centre, Massey University Manawatu (Turitea), Tennent Drive, Palmerston North 4474, New Zealand

**Keywords:** 405 nm blue light, far-UVC, ultraviolet light, feline infectious peritonitis virus, viral inactivation, light disinfection, coronavirus, pandemic

## Abstract

Transmission of the severe acute respiratory syndrome coronavirus 2 (SARS-CoV-2) occurs through respiratory droplets passed directly from person to person or indirectly through fomites, such as common use surfaces or objects. The aim of this study was to determine the virucidal efficacy of blue LED (405 nm) and far-UVC (222 nm) light in comparison to standard UVC (254 nm) irradiation for the inactivation of feline infectious peritonitis virus (FIPV) on different matrices as a model for SARS-CoV-2. Wet or dried FIPV on stainless steel, plastic, or paper discs, in the presence or absence of artificial saliva, were exposed to various wavelengths of light for different time periods (1–90 min). Dual activity of blue LED and far-UVC lights were virucidal for most wet and dried FIPV within 4 to 16 min on all matrices. Individual action of blue LED and far-UVC lights were virucidal for wet FIPV but required longer irradiation times (8–90 min) to reach a 4-log reduction. In comparison, LED (265 nm) and germicidal UVC (254 nm) were virucidal on almost all matrices for both wet and dried FIPV within 1 min exposure. UVC was more effective for the disinfection of surfaces as compared to blue LED and far-UVC individually or together. However, dual action of blue LED and far-UVC was virucidal. This combination of lights could be used as a safer alternative to traditional UVC.

## 1. Introduction

In January 2020, the World Health Organization (WHO) disseminated news about identification of a novel coronavirus associated with a cluster of 41 cases of viral pneumonia with 7 severe cases and 1 death in Wuhan, China [1]. Severe acute respiratory syndrome coronavirus 2 (SARS-CoV-2) has since then spread across the globe causing a worldwide pandemic with over 169 million confirmed cases and over 3.5 million recorded deaths as of the 28th of May 2021 [2,3,4].

Coronaviruses are enveloped viruses with a positive sense non-segmented RNA genome that infect a variety of vertebrate hosts including humans. Coronaviruses can be classified into 39 species, 27 subgenera, 5 genera and 2 subfamilies within the family *Coronaviridae*, suborder *Cornidovirineae*, order *Nidovirales*, and realm *Riboviria* [5]. Coronavirus infections have been associated with a variety of clinical outcomes among animals, ranging from mild respiratory or gastrointestinal diseases to fatal systemic diseases [6]. One example of the latter is feline infectious peritonitis (FIP) which is caused by FIP virus (FIPV), a virulent pathotype of feline coronavirus, an alphacoronavirus with worldwide distribution. Up to 100% of cats acquire feline coronavirus infection within the first year of life [7,8]. Occasionally, the virus mutates within an individual cat, which leads to the transition from a self-limiting, subclinical, or mild gastrointestinal disease to a fatal systemic disease termed FIP [7].

Humans are hosts to four respiratory coronaviruses including two alphacoronaviruses (HCoV-229E and HCoV-NL63) and two betacoronaviruses (HCoV-HKU1 and HCoV-OC43) that are endemic worldwide [9]. Infections with these endemic viruses are typically associated with mild to moderate respiratory disease, often referred to as “common colds” [10]. Three additional betacoronaviruses have emerged in human populations in the past two decades: SARS-CoV-1 [11,12], Middle East respiratory syndrome coronavirus (MERS-CoV) [13], and the current pandemic virus SARS-CoV-2 [5]. In contrast to generally self-limiting upper respiratory tract infections associated with endemic human coronaviruses, a comparatively high proportion of people infected with these three emerging coronaviruses develop severe pneumonia with estimated case fatality rates ranging from up to 5% for SARS-CoV-2 [14] to 35% for MERS-CoV [15]. While SARS-CoV-1 has not been detected among humans since 2004, and transmission of MERS-CoV has been geographically confined to the Middle East, SARS-CoV-2 has spread throughout the world within the first half a year of its emergence in China, resulting in a global health crisis [16].

All three emerging human coronaviruses are believed to be of zoonotic origin. In a recent study, a total of 73 coronaviruses were identified among 1067 bats from 21 species in 3 provinces of China, with an overall prevalence of 6.8% [17]. The 41 betacoronaviruses were all closely related to the current members of the species *severe acute respiratory syndrome related viruses*, including SARS-CoV-1 and SARS-CoV-2, highlighting the likely role of bats as direct or indirect source not only for SARS-CoV-1 [18], MERS-CoV [19], and most likely SARS-CoV-2 [20], but also for other coronaviruses with the potential to pose a threat to human health. Continued encroachment of humans into wildlife habitats linked to deforestation and urbanization, as well as the ease and speed of worldwide travel and trade, are likely to facilitate any future spillover events that may lead to pandemics [21].

Direct transmission of SARS-CoV-2 occurs by respiratory droplets during coughing, sneezing, or talking. Transmission can also occur through direct or indirect contact with body fluids/secretions, for example saliva or feces [22,23,24]. Indirect transmission is thought to occur through fomites such as common-use surfaces or contaminated objects. Viable SARS-CoV-2 has been detected up to four days after contamination of various surfaces, with the length of survival dependent on the environmental conditions and the type of surface matrix [25,26,27].

The ability of SARS-CoV-2 to remain viable on surfaces and the evidence for indirect transmission highlights the need for effective surface sanitization in high-risk environments such as hospitals, hotels, or airports where there are large numbers of people at any one time. According to the current literature there is no evidence of foodborne transmission of SARS-CoV-2 since cooking or pasteurization inactivates the virus, and the risk of transmission through the outside packaging of food products is considered to be low [28,29]. Alcohol and chemical disinfectants can be used to inactivate SARS-CoV-2 [30]. However, the use of chemical disinfectants on a regular basis over prolonged periods of time may affect the integrity of materials (for example, those used for personal protective equipment (PPE) or surfaces and equipment in public places).

The interest in the use of ultraviolet (UV) light (300–400 nm) as an alternative to chemical sanitizers for the disinfection of surfaces, air, and PPE has increased substantially in the last decade [31]. UV lights are also commonly used in the food industry to lower the microbial load on packaging materials, as well as on food products themselves. UV radiation has been classified into three groups depending on its effects on living creatures: UVA (320–400 nm), UVB (290–320 nm), and UVC (200–290 nm) [32]. UVC radiation has shown the greatest antimicrobial activity with the maximum absorption of nucleic acids at 265 nm and is commonly used in germicidal mercury lamps at 254 nm [33]. However, due to the harmful properties of UVC radiation to human health through cellular damage to the skin and eyes, the use of these lamps in areas that are heavily occupied by people is not considered safe and is therefore only recommended in closed box systems [34].

Light at other wavelengths including far-UVC (222 nm) and blue LED (405 nm) has also been shown to have some antimicrobial activity with potentially less harmful effects on human tissues [35,36]. The mechanism of virucidal activity of far-UVC krypton chloride (Kr-Cl) excimer lamps is similar to that of germicidal mercury lamps. Depending on the virus, the radiation may mutagenize the viral genome or damage viral structural components such as capsid binding proteins [37]. The main difference between the two lights is their penetrability, where far-UVC is unable to penetrate through thick cell layers (stratum corneum) of the skin or the tear layers within the eye [38]. Blue LED light (400 to 450 nm) has a different mechanism of cellular damage, which relies on creation of reactive oxygen species (ROS) due to the absorption of the light by photosensitizers, which leads to their activation. Reactive oxygen species can target viral structural (e.g., receptors) or nonstructural (e.g., viral enzymes) proteins or induce nucleic acid damage through the oxidation of guanosine residues [39].

The aim of the current study was to investigate the effect of blue LED (405 nm) and far-UVC (222 nm) lights used individually or in combination on viability of FIPV as a surrogate for SARS-CoV-2, and to compare the efficacy of these two light sources at inactivating FIPV with the efficacy of LED UVC (265 nm) and standard germicidal mercury bulbs (254 nm).

## 2. Materials and Methods

### 2.1. Cells

Crandell-Rees feline kidney (CRFK) cells, were obtained from the American Tissue Culture Collection (ATCC) and maintained using standard protocols [40] in growth medium (GM), which comprised Advanced Dulbecco′s modified Eagle medium (DMEM, ThermoFisher Scientific, Waltham, MA, USA) supplemented with 2% fetal calf serum (ThermoFisher Scientific, Waltham, MA, USA), 1% antibiotic solution (Penstrep, ThermoFisher Scientific, Waltham, MA, USA) containing 10,000 unit/mL of penicillin and 10,000 µg/mL of streptomycin, and 1% Glutamax (ThermoFisher Scientific, Waltham, MA, USA). Cells were maintained at 37 °C in a humidified atmosphere with 5% CO_2_.

### 2.2. Preparation of Virus Stock

A tissue culture adapted strain of FIPV (WSU 79-1146, sourced from ATCC) was propagated and titrated in CRFK cells using standard virological methods [41]. The flasks inoculated with the virus were freeze-thawed at −80 °C/37 °C when 80 to 100% of the monolayer showed viral cytopathic effect (CPE). The cell lysates were clarified by low-speed centrifugation at 300× *g* for 5 min, aliquoted and stored at −80 °C until further use. One aliquot was removed from the freezer and titrated to determine the titer of the entire batch. The titer of the prepared virus ranged from 3.56 × 10^5^ to 1.12 × 10^7^ depending on the batch.

### 2.3. Virus Titration

Virus titrations were performed using standard virological methods. Briefly, serial 10-fold dilutions of the virus in GM (50 µL/well) were made across a 96-well tissue culture plates (Nunc, ThermoFisher Scientific, Waltham, MA, USA) in triplicate and CRFK cells (1:5 split) were then added to each well. Cell culture control (cells maintained in GM without the addition of the virus) and virus control (FIPV of a known titre) were included with each titration. The plates were assessed for the presence of viral CPE after four days of incubation at 37 °C in a humidified atmosphere with 5% CO_2_. The titers were calculated using the method of Spearman-Kärber [42] and presented as tissue culture infective dose 50% (TCID_50_) with standard deviation (SD). Samples that showed no CPE during titration were subjected to large volume culture. This was achieved by absorbing 0.5 mL of each sample onto 90% confluent monolayer of CRFK cells in a 12-well tissue culture dish for 30 min. The inoculum was then replaced with GM and the plate incubated at 37 °C in 5% CO_2_ for five days (virus passage 1). The plates were then freeze-thawed, and 0.5 mL of the cell lysate from each well was transferred into a well of a new 12-well plate with CRFK cells at the time of seeding (virus passage 2). The samples were considered negative for FIPV if no CPE was present in any of the passages (log titre of ≤0.3 TCID/mL), and positive for FIPV if viral CPE was observed after either the first or the second passage (log titre of 0.8 TCID/mL).

### 2.4. Virus Contamination and Recovery Matrices

Three different matrices were used for virus contamination: stainless steel (metal) type 304 (diameter 20 mm: AgResearch Lincoln, Christchurch, New Zealand), food-grade polyoxymethylene copolymer plastic (diameter 20 mm; DOTMAR Ltd., Auckland, New Zealand) (plastic) and filter paper (diameter 20 mm; Lab Supply Ltd., Dunedin, New Zealand) (paper) discs. Sterile autoclaved discs were placed in 12-well tissue culture plates (one disc per well) in triplicate. An aliquot (100 µL) of FIPV of known titer was then spread on top of each disc and the plate was left in the biosafety class II cabinet at room temperature until the virus dried onto the surface (approximately 1.5 h).

To recover the virus from metal and plastic discs, 200 µL of GM was applied to the surface of each disc on top of the dried virus. The plates were placed on ice for 20 min allowing the virus to rehydrate. An additional 800 µL of GM was then added to each well and the excess media was used to wash the discs by pipetting up and down until the outline of the dried virus was no longer visible. The contents of each well were then transferred to individual cryovials and placed on ice for titrations on the same day. For the recovery of the virus from paper discs, 1 mL of GM was applied to each disc, and the plates were shaken at 200 rpm for 30 min before the contents of each well was transferred to individual cryovials as described above for metal and plastic. Discs contaminated with media only (no virus) were used as negative controls. An aliquot of the stock virus used for disc contamination was kept on ice throughout the experiment and titrated as a positive virus control.

The virus was spotted onto the discs either by itself or in the presence of artificial saliva (NaHCO_3_ 5.2 g/L, NaCl 0.88 g/L, K_2_HPO_4_ 1.36 g/L, KCL 0.48 g/L, α amylase 2000 units/L, porcine gastric mucin 2 g/L [36]. This was achieved either by dilution of the virus 1:1 in saliva immediately before 100 µL of the virus /saliva mix was applied to the disc or by depositing 100 µL of the virus onto discs containing dried saliva. The discs with the dried saliva were prepared by spotting 200 µL of artificial saliva onto the surface of each disc and allowing it to dry overnight in the biosafety class II cabinet.

### 2.5. Light Sources

Three different custom-made light emitting units were used in this study: (1) Violeta 1.0 (EnergyLine Ltd., Christchurch, New Zealand) light unit with three blue LED 405 nm bars (total radiant flux = 36 W) and two LED UVC 265 nm light bars (total radiant flux = 21.5 mW/cm^2^ at 20 mm from lamp surface); (2) Violeta 2.0 (EnergyLine Ltd., Christchurch, New Zealand) light unit with three blue LED 405 nm bars (total radiant flux = 36W) and four 12 W USHIO far-UVC 222 nm mercury-free excimer lamps (total radiant flux = 21.5 mW/cm^2^); and (3) Violeta 3.0 (EnergyLine Ltd., Christchurch, New Zealand) light unit with four blue LED 405 nm bars (total radiant flux = 48 W), two 36 W UVC 254 nm lamps and one 60 W HO UVC 254 nm lamp (total radiant flux = 43 W). The arrays were equipped with heat sinks and fans to minimise heat transfer to the virus samples. To determine the best distance and position of the test samples for light treatment, spatial data was collected for light intensity (Figure S1). All light sources were placed 25 cm above the surface of the plates giving an average irradiance of 1686 µW/cm^2^ for 265 nm, 322.7 µW/cm^2^ for 222 nm, 1947 µW/cm^2^ for 2 × 36 W 254 nm bulbs and between 16,819.9 and 20,853 µW/cm^2^ for 405 nm bulbs. All irradiance measurements were performed with a SpectriLight ILT950 Spectroradiometer (International Light Technologies, Peabody, MA, USA).

### 2.6. Temperature and Humidity Readings

Temperature and humidity were measured during each light exposure trial using an Tinytag Plus 2 TGP-4500 data logger. The surface temperature was measured using a Fluke Model 52 thermometer (Global Test Supply, Wilmington, NC, USA). The average temperature and humidity were taken over a 90-min exposure period to assess any changes during light treatment for individual (222 nm and 405 nm) and dual (222 + 405 nm) light in three independent experiments (Figure S2A,B).

### 2.7. Light Exposure Trials

#### 2.7.1. Effects of Light Exposure on Viability of Virus in Suspension

A 500 µL aliquot of virus suspension was pipetted into each of the 6 wells (A, B, and C in columns 1 and 5) of the 24-well plate and a 500 µL aliquot of media was pipetted into each of two wells (D, columns 1 and 5) of the same plate as negative controls. Half (column 1) of the 24-well plate was exposed to light (treated wells) and the second half (column 5) was covered with a strip of black paper to prevent exposure to the light (untreated controls). Replicate plates were prepared for each time point. The plates were placed inside a light box and treated with light for the pre-determined time, after which the content of each well was transferred to a cryovial. The vials were kept on ice until titrated on the same day, and the remaining sample was stored at −80 °C.

#### 2.7.2. Effects of Light Treatment on Viability of Virus on Different Surface Matrices

Sterile metal, plastic, or paper discs were placed in wells of 12-well plates in triplicate and contaminated with FIPV with or without addition of artificial saliva as described in Section 2.4. In some experiments the virus spotted on the discs was subjected to the light treatment without allowing it to dry (wet virus). Duplicate plates were prepared for each time-point, one of which was covered with black paper (untreated virus control). Both covered and uncovered plates were exposed to light for the pre-determined time. At each time point, one treated and one control plate were removed from the light box. The dried virus was recovered from the discs as described in Section 2.4. For the wet virus the method was adjusted so that 100 µL of GM was used for virus recovery from metal and plastic discs and 900 µL for filter paper discs in order to keep the final volume at 1 mL. Titrations were carried out on the same day.

### 2.8. Data and Statistical Analysis

The titers with SD were calculated using an online calculator [43]. The log reduction was calculated by subtracting the log titre of the virus recovered from the treated samples from the log titer of the corresponding untreated controls. For calculation of the log titer reduction, samples that were negative on titration but produced CPE during large volume culture were considered to have a log titer of 0.8, and samples that were negative on large volume culture were considered to have a log titer of 0.3. The treatment was considered effective at inactivating FIPV if either a ≥4-log reduction was observed between treated and control samples or there was no recovery of infectious virus from the treated sample after the large volume culture, even if the log reduction was lower than 4. Averages and standard deviation for graphical and tabular representation were performed using GraphPad Prism version 9.1.2 for windows, GraphPad Software, San Diego, CA, USA, www.graphpad.com. Statistical analysis was completed using two-way ANOVA and post hoc analysis using Tukeys test (GraphPad Prism) was used to compare the difference between light wavelengths and differences between the samples that were dried or wet with or without the addition of artificial saliva.

## 3. Results

### 3.1. Inactivation of FIPV after Exposure to UVC LED and UVC Mercury Lights

To assess if there was a difference between LED UVC (265 nm) and standard mercury UVC lamps (254 nm) in inactivating FIPV, metal, plastic, and paper matrices inoculated with either wet or dried FIPV in the presence or absence of artificial saliva were exposed to a comparable intensity of light (1686 µW/cm^2^ for 265 nm and 1947 µW/cm^2^ for 254 nm) for 1-min. Infectious FIPV was not recovered from any of the matrices after treatment of the wet virus with light at either 265 nm or 254 nm, with at least a 4-log reduction in titer observed under all test conditions (Table 1). Treatment of the dried virus with individual or dual 265 nm + 405 nm lights was virucidal under most, but not all, testing conditions, with the poorest virucidal effect exhibited by 265 nm light against the virus spotted on plastic and paper (Table 1).

### 3.2. Inactivation of FIPV in Liquid Suspension after Exposure to Light at 405 nm and 222 nm

**Figure 1:**
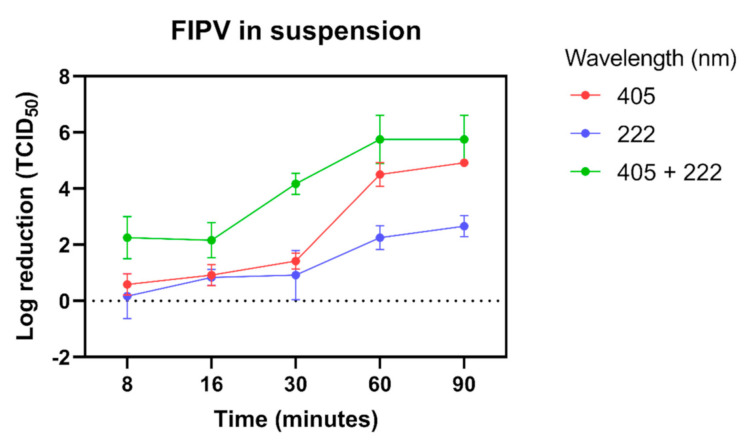
Inactivation of feline infectious peritonitis virus (FIPV) in growth media by blue LED 405 nm, far-UV 222 nm and dual 405 nm + 222 nm lights. All exposures were done at 25 cm distance. Error bars represent standard deviation.

Exposure of FIPV in liquid suspension to dual 405 nm + 222 nm lights resulted in a 4-log reduction in titre within 30-min of exposure (Figure 1). In comparison, FIPV exposed to individual 222 nm or 405 nm lights showed only a 1-log reduction in titre after 30-min. Although the log reduction increased with longer exposure to individual lights for both 222 nm and 405 nm, the combined effect of two lights was always stronger than the effect of each individual light, with approximately a 6-log reduction observed after a 60- and 90-min exposure (Figure 1). This level of reduction was 1 to 2 logs higher compared with exposure to 405 nm light alone, and 3 to 4 logs higher compared with exposure to 222 nm light alone.

### 3.3. Inactivation of FIPV on Different Matrices after Exposure to Lights at 405 nm and 222 nm

**Figure 2:**
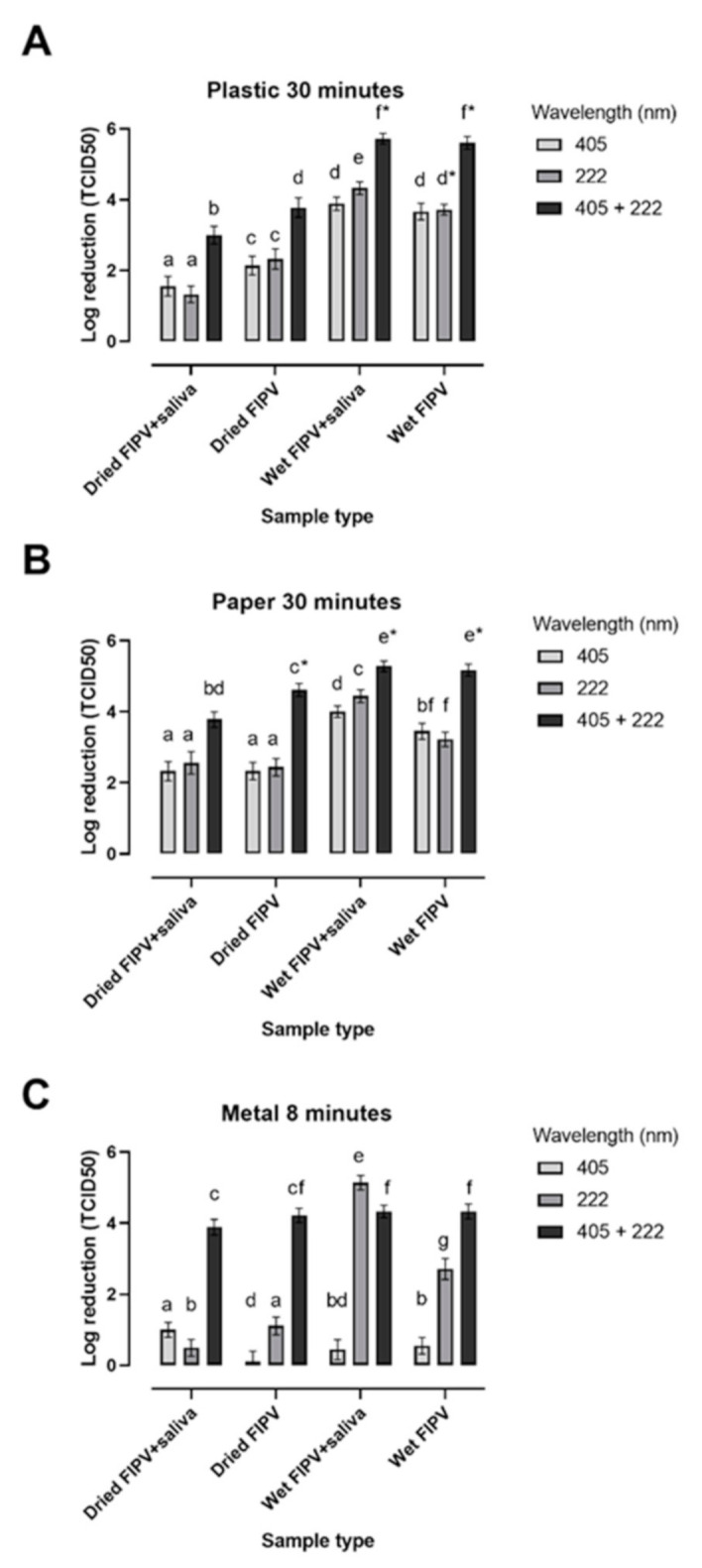
Log reduction of feline infectious peritonitis virus (FIPV) spotted on contaminated surfaces and exposed to blue LED (405 nm), far-UVC (222 nm) and dual (405 nm + 222 nm) lights. FIPV was either dried onto plastic (**A**), paper (**B**), or metal (**C**) discs in the presence or absence of saliva or left wet prior to being exposed to light for 30 min (plastic and paper) or 8 min (metal). All exposures were done at 25 cm distance. Data is displayed as mean log reduction in titre ± SD. Statistically significant differences are indicated by different letters (a to f), (*p* < 0.05). * No CPE observed after large volume culture. TCID_50_ = tissue culture infectious dose, 50%.

In general, the exposure to dual lights (405 nm + 222 nm) resulted in a higher log reduction than exposure to individual lights for the same time under most testing conditions (Figure 2).

Dual light was virucidal for wet FIPV on metal discs after an 8 min exposure with or without artificial saliva, and for the dried virus without saliva (Figure 2C). A slightly lower than a 4-log reduction (3.89 logs) was observed for the dried virus in the presence of saliva. In contrast, there was no inactivation of the dried virus treated with individual lights for the same length of time, with ≤1-log reduction in titre observed for either 405 nm or 222 nm lights, showing that dual exposure produced a synergistic effect. The ability of individual lights to inactivate wet FIPV within 30-min was light-dependent with only ≤1-log reduction observed after exposure to 405 nm light, but a 5- and 2-log reduction for samples with and without saliva, respectively, exposed to 222 nm light (Figure 2C).

A 30 min exposure to dual light was virucidal for wet FIPV on plastic discs, with a 5-log reduction observed with or without artificial saliva, but not effective at killing the dried virus, with a 3-log reduction in titre and recovery of infectious virus (Figure 2A). Exposure for the same length of time to each individual light resulted in lower reduction levels, which were similar for 222 nm and 405 nm and ranged from 1 to 2 logs for dried FIPV and from 3 to 4 logs for wet FIPV (Figure 2A).

By 60-min, exposure of the wet virus to either 222 nm or 405 nm lights were virucidal on all matrices tested, as indicated by the lack of recovery of any infectious virus or a >4-log reduction in titre (Table 2). The wet virus exposed to dual 405 nm + 222 nm lights was fully inactivated within 4 to 16 min (Table 2). Both for 222 nm light and for dual lights, samples with artificial saliva tended to become inactivated within a shorter time than those without saliva (Table 2).

In contrast, the highest reduction in titer was still only 2 to 3 logs after a 90-min exposure of dried FIPV to either 222 nm or 405 nm lights, indicating that neither 222 nm nor 405 nm light alone was effective at inactivating the dried virus (Table 2). We were unable to assess if log reduction increased after exposure of the dried virus to 405 nm + 222 nm lights for longer than 30-min, as viability of the control virus declined. However, the live virus was not recovered following exposure to dual 405 nm + 222 nm lights at 60- and 90-min time points.

## 4. Discussion

The impact of the 2020 SARS-CoV-2 pandemic has highlighted the need for effective countermeasures to control the spread of the virus through surface and object contamination, particularly in public areas. This has driven the need for disinfection systems that can inactivate SARS-CoV-2 effectively and safely. In this study we aimed to determine the viricidal potential of far-UVC (222 nm) and blue LED (405 nm) lights when used individually or together. We also compared these wavelengths with UVC LED (265 nm) and mercury germicidal lamps (254 nm) that have been traditionally used for disinfection purposes.

Due to the serious health risks associated with working with SARS-CoV-2, it requires high levels of biosafety containment which is achievable only at selected laboratories [44]. Hence, the work presented in this paper was performed with FIPV, which was used as a surrogate/model virus for SARS-CoV-2. According to the FDA guidelines [45], a model virus is defined as “a virus which is closely related to the known or suspected virus (same genus or family), having similar physical and chemical properties to the observed or suspected virus”. Additional points to consider in selection of the appropriate model viruses include their ability to be grown to a high titer in a suitable cell culture system and availability of reliable assays for detection of such growth [45]. Both SARS-CoV-2 and FIPV are classified within the same family *Coronaviridae*, but within different genera: *Betacoronavirus* for SARS-CoV-2 and *Alphacoronavirus* for FIPV. All coronaviruses share similar structure and genome replication strategies, with classification into different genera based on phylogenetic analysis and less than 46% sequence identity in the conserved replicase domains [46]. Except for the relatively close phylogenetic relationships between different betacoronaviruses, the only general characteristic that sets them apart from other coronaviruses is their non-structural protein 1 (nsp1), which is distinct in size and sequence from nsp1 of alphacoronaviruses and does not have an equivalent in the gammacoronaviruses [46]. As such, any coronavirus that can be grown in cell culture to a relatively high titer, such as FIPV, should be suitable as a model virus for the in vitro work described in this paper. This is supported by the fact that no significant differences were found in virucidal efficacy of selected chemical disinfectants, heating, and ultraviolet radiation against canine coronavirus (an alphacoronavirus) and MHV (a betacoronavirus) [47]. Consequently, feline coronavirus has been used by others as a surrogate for human betacoronaviruses including SARS-CoV-2 [48,49].

A variety of animal coronaviruses including infectious bronchitis virus (an avian deltacoronavirus) or transmissible gastroenteropathy virus (an alphacoronavirus of pigs) have been used as surrogates for human coronaviruses including SARS-type viruses (reviewed by [50]). While the data obtained using a surrogate virus should provide a useful approximation of the expected effects against a target virus, there are inherent limitations to the use of surrogate viruses. Similar limitations also apply to the use of the target virus, since the data obtained with a selected laboratory strain of the target virus may not fully represent a variety of field viruses and environmental conditions outside of the laboratory. To exemplify this, the titre of SARS-CoV was reduced by >4-logs within 6 min of exposure to UVC (254 nm) from a 3 cm distance with no infectious virus recovered at 15 min time-point [51]. However, addition of 10 to 25% of bovine serum albumin to the virus suspension resulted in the increase of stability of the virus, with little loss in titre after a 1 h exposure to the UVC light under the same conditions [52]. Others reported a drop in the levels of SARS-CoV after a 15 min exposure to UV light (260 nm) from an 80 cm distance, with full inactivation of the virus within 60 min [53]. Unfortunately, the virus was not titrated in that study and the levels were assessed based on the extent of cytopathic effect after 48 h of culture. Consequently, the results presented in the current study should be treated as approximation of the expected effects of various lights and exposure conditions on survival of SARS-CoV-2.

We have shown that the virucidal effects of LED UVC (265 nm) light is comparable to that of 254 nm germicidal lamps. The UVC (254 nm) technology has been widely used for surface disinfection in the food and medical industries [54,55,56]. The advantage of LED technology compared to standard mercury lamps is the ability of LED bulbs to warm up quickly and function effectively at broad ranges of temperatures including refrigeration temperatures (−1.5 °C to 4 °C), as well as their increased safety (since they do not contain mercury). Due to the glass casing of the mercury lamp with 254 nm, lights are prohibited in food production areas as glass is a food safety hazard. In contrast, UVC LED casings are made from plastic and could therefore be used in the food production environment.

The virucidal effect of dual exposure to 222 nm + 405 nm lights was greater than the effect of each individual light alone within the same time frame. The “additive effect” has been observed in most dual wavelength light studies where the second law of photochemistry dictates that “each photon can (at most) cause the photochemical reaction of just one light-absorbing molecule” [57]. Thus, the inactivation achieved by combined treatment is the same as sum of the inactivation achieved by each treatment. While for some matrices and time points the combined effect of two lights in the current study appeared additive (e.g., virus in suspension at 60 min or dried FIPV on paper at 30 min), the log inactivation following exposure to dual light was typically lower than the sum of log reduction for individual lights, suggesting that some limitations to the truly additive effect exist. The virucidal effect of dual light exposure of the dried virus spotted on metal was far greater than the sum of the log virus reductions observed after exposure to individual lights at 8 min time point (Figure 2). The virus inactivation by dual light on metal discs was also faster than that on plastic and paper surfaces, as it was for a 222 nm light, but not for 405 nm light (Table 2). One possible explanation for these effects is the high thermal conductivity of the metal [58]. Heat can have a direct effect on survival of viruses. In addition, heat elevation has been found to induce ROS production in bacteria [59]. Both mechanisms could theoretically explain the increase in rate of inactivation of FIPV on metal discs compared to paper or plastic discs It remains unclear, however, why these surface-related effects were apparent for 222 nm and combined 222 nm + 405 nm, but not for 405 nm light.

In general, the wet virus was more susceptible to inactivation by 222 nm and 405 nm light than the virus dried onto the surfaces within the first 8 to 30 min of exposure (Figure 2). The extent of this effect varied between surfaces and lights, with the most apparent difference observed between wet and dried FIPV spotted on metal discs exposed to 222 nm light. One possible explanation for this resistance to light inactivation of dried virus as compared with the wet one is the presence of a residue layer of dried GM that may have provided some protection against the light exposure [60]. The far-UVC (222 nm) light has limited penetration depth compared with traditionally used germicidal UV light (254 nm or 265 nm) because it is strongly absorbed by proteins and other biomolecules [61,62]. The fact that both dried and wet FIPV exposed to 254 nm or to 265 nm light was fully inactivated within minutes (Table 1) supports this view.

As the penetration depth increases with increased wavelength [63], the same cannot explain the differences in virucidal activity of 405 nm light following a 30 min exposure of dry and wet virus on plastic and paper discs, as well as lack of efficacy at inactivating both the wet and dried virus within 8 min on metal discs compared with that of 222 nm light. The inactivation by 405 nm LED relies on the presence of photosensitizers, which become excited in the presence of oxygen upon emitting blue light. The excited photosensitizers produce ROS, which are responsible for damage to structural components of pathogens including proteins, lipids, and nucleic acids [64,65]. Our data suggest that drying affects these processes and diminishes the virucidal activity of the 405 nm light, although the exact mechanisms of such interactions still need to be elucidated.

The recovery of the dried control virus from all three matrices declined with time, making the assessment of the influence of light on virus inactivation at longer time-points difficult. The sensitivity of coronaviruses to desiccation has been described by others, with rapid loss of infectivity of HCV-229E and HCV-OC43 within one to three hours of drying at room temperature on various surfaces [66]. The unavoidable increase in temperature (Figure S2A) and decrease in humidity (Figure S2B) with increased time of exposure was likely to further facilitate loss of FIPV viability at longer time-points.

The presence of saliva did not have a detrimental effect on the virucidal activity of blue LED (405 nm) and far-UVC (222 nm) lights on any of the surfaces tested, with a similar log reduction observed for samples with and without saliva except for wet FIPV spotted on metal (Figure 2). For the latter, the addition of saliva seemed to facilitate the virucidal activity of the far-UVC light. The lack of interference of artificial saliva with virus inactivation by blue LED and far-UVC lights is in contrast to microbial inactivation by most chemical disinfectants and the traditional UVC light, which show decreased efficacy in the presence of organic matter [67]. Occasionally, the presence of saliva seemed to facilitate virucidal activity of light as evidenced by a shorter time needed for virus inactivation under the same conditions for samples with and without saliva (Table 2). As this effect was most apparent for 222 nm and combined 222 nm + 405 nm lights, it was unlikely to be explained solely by the presence of photosensitisers, such as mucin, in the artificial saliva that can be readily oxidized to produce ROS (as previously shown by Tomb et al. (2017)). If that were true, we would have expected to see the greatest effect of artificial saliva on the speed or level of FIPV activation in samples exposed to 405 nm light, which was not the case. However, artificial saliva has also been shown to facilitate UVB light inactivation of SARS-CoV-2, possibly through the production of toxic intermediates generated in the reaction [60,68]. Similar mechanisms may contribute to the increased inactivation of FIPV by far-UVC (222 nm) light in the presence of saliva, but these still need to be determined.

This work shows the potential for light technology with dual 405 nm and 222 nm lights for the disinfection of surfaces contaminated with coronaviruses in areas where traditional UVC light may not be applicable due to health concerns or for disinfection of materials that may deteriorate following repeated exposure to the UVC light. While UVC LED (265 nm) and traditional mercury lamps (254 nm) may be preferable in a closed-box system due to their high efficacy at inactivating FIPV within minutes of exposure, the combined 405 nm + 222 nm light may be preferable in public areas such as airports, shopping centres or hospital rooms. The necessity for slightly longer exposure times as compared with UVC is balanced by the potential to use this technology in large spaces, possibly in the presence of people or animals. In addition, the increased virucidal activity of combined 222 nm + 405 nm light in the presence of artificial saliva provides an important advantage under field conditions, where prior cleaning of the materials/surfaces may not be practical.

Although the motivation for this work was the desire to investigate the potential of light to inactivate SARS-CoV-2, the results are also relevant for other human and animal coronaviruses. Specifically, light disinfection may be considered in catteries and animal shelters to minimize environmental contamination with coronaviruses and hence, to help control coronavirus-associated diseases including FIP.

Future work could include the investigation of the effect of pulsing each light in quick succession on the ability of the individual and dual 405 nm + 222 nm light to inactivate FIPV and other coronaviruses, as well as the ability of this light combination to inactivate enveloped and non-enveloped viruses from other families.

## Figures and Tables

**Table 1:** Time and dose at which a 4-log reduction in titer was reached for feline infectious peritonitis virus (FIPV) in media as well as for dried or wet virus spotted on three different surfaces (metal, paper, plastic) in the presence and absence of artificial saliva, after exposure to UVC light at 254 nm, 265 nm, or to dual 265 nm + 405 nm light. When a 4-log reduction was not observed, the highest log reduction achieved is shown. The light was placed at a distance of 25 cm from the virus.

Matrix/Virus	Saliva	Time (min)	Dose (J/cm^2^)	Control Virus (TCID_50_/mL ± SD)	Treated Virus (TCID_50_/mL ± SD)	Log Reduction ± SD
**265 nm + 405 nm**						
Media	−	1	1.350	5.13 ± 0.17	0.30 ± 0.00	4.83 ± 0.17 ^1^
Metal						
Wet	+	1	1.350	5.58 ± 0.20	0.30 ± 0.00	5.28 ± 0.20 ^1^
Wet	−	1	1.350	5.80 ± 0.21	0.91 ± 0.11	4.89 ± 0.24
Dried	+	1	1.350	5.25 ± 0.18	0.30 ± 0.00	4.94 ± 0.18 ^1^
Dried	−	1	1.350	5.91 ± 0.18	1.25 ± 0.18	4.67 ± 0.25
Paper						
Wet	+	1	1.350	5.58 ± 0.20	0.30 ± 0.00	5.28 ± 0.20 ^1^
Wet	−	1	1.350	6.13 ± 0.17	0.80 ± 0.00	5.33 ± 0.17 ^2^
Dried	+	1	1.350	5.13 ± 0.17	0.30 ± 0.00	4.83 ± 0.17 ^1^
Dried	−	1	1.350	5.02 ± 0.15	0.30 ± 0.00	4.72 ± 0.15 ^1^
Plastic						
Wet	+	1	1.350	5.58 ± 0.15	0.30 ± 0.00	5.28 ± 0.15 ^1^
Wet	−	1	1.350	5.25 ± 0.18	0.30 ± 0.00	4.94 ± 0.18 ^1^
Dried	+	1	1.350	3.91 ± 0.11	0.30 ± 0.00	3.61 ± 0.11 ^1^
Dried	−	1	1.350	4.80 ± 0.16	0.80 ± 0.00	4.00 ± 0.16 ^2^
**265 nm**						
Media	−	1	0.101	5.25 ± 0.18	0.30 ± 0.00	4.94 ± 0.18 ^1^
Metal						
Wet	+	1	0.101	5.69 ± 0.11	0.30 ± 0.00	5.39 ± 0.11 ^1^
Wet	−	1	0.101	4.91 ± 0.18	0.30 ± 0.00	4.61 ± 0.18 ^1^
Dried	+	1	0.101	3.25 ± 0.20	0.30 ± 0.00	2.94 ± 0.20 ^1^
Dried	−	1	0.101	3.91 ± 0.11	0.30 ± 0.00	3.61 ± 0.11 ^1^
Paper						
Wet	+	1	0.101	5.69 ± 0.18	0.30 ± 0.00	5.39 ± 0.18 ^1^
Wet	−	1	0.101	4.58 ± 0.20	0.30 ± 0.00	4.28 ± 0.20 ^1^
Dried	+	1	0.101	4.80 ± 0.21	0.91 ± 0.11	3.89 ± 0.24 ^2^
Dried	−	1	0.101	5.13 ± 0.21	0.30 ± 0.00	4.83 ± 0.21 ^1^
Plastic						
Wet	+	1	0.101	5.91 ± 0.11	0.30 ± 0.00	5.61 ± 0.11 ^1^
Wet	−	1	0.101	4.69 ± 0.18	0.30 ± 0.00	4.39 ± 0.18 ^1^
Dried	+	1	0.101	4.02 ± 0.15	0.80 ± 0.00	3.22 ± 0.15 ^2^
Dried	−	1	0.101	4.91 ± 0.18	1.58 ± 0.24	3.33 ± 0.17 ^2^
**254 nm**						
Media	−	1	0.117	7.13 ± 0.17	0.91 ± 0.11	6.22 ± 0.20
Metal						
Wet	+	1	0.117	5.80 ± 0.21	0.30 ± 0.00	5.50 ± 0.21 ^1^
Wet	−	1	0.117	5.69 ± 0.11	0.30 ± 0.00	5.39 ± 0.11 ^1^
Dried	+	1	0.117	4.02 ± 0.15	0.30 ± 0.00	3.72 ± 0.15 ^1^
Dried	−	1	0.117	4.80 ± 0.00	0.30 ± 0.00	4.50 ± 0.00 ^1^
Paper						
Wet	+	1	0.117	5.47 ± 0.21	0.30 ± 0.00	5.17 ± 0.21 ^1^
Wet	−	1	0.117	5.69 ± 0.11	0.30 ± 0.00	5.39 ± 0.11 ^1^
Dried	+	1	0.117	5.02 ± 0.15	0.80 ± 0.00	4.22 ± 0.15 ^2^
Dried	−	1	0.117	4.47 ± 0.17	0.30 ± 0.00	4.17 ± 0.17 ^1^
Plastic						
Wet	+	1	0.117	5.25 ± 0.18	0.30 ± 0.00	4.94 ± 0.18 ^1^
Wet	−	1	0.117	5.47 ± 0.17	0.30 ± 0.00	5.17 ± 0.17 ^1^
Dried	+	1	0.117	4.36 ± 0.18	0.30 ± 0.00	4.05 ± 0.18 ^1^
Dried	−	1	0.117	4.13 ± 0.17	0.30 ± 0.00	3.83 ± 0.17 ^1^

^1^ Log reduction was acquired by large volume culture, no infectious virus detected. ^2^ Log reduction was acquired by large volume culture.

**Table 2:** Time and dose at which a 4-log reduction in titer was reached for dried or wet feline infectious peritonitis virus (FIPV) on three different surfaces (metal, paper, plastic) in the presence and absence of artificial saliva. All exposures were conducted at 25 cm distance.

Matrix/Virus	Saliva	Time (min)	Dose (J/cm^2^)	Control Virus (TCID_50_/mL ± SD)	Treated Virus (TCID_50_/mL ± SD)	Log Reduction ± SD
**405 nm + 222 nm**						
Metal						
Wet	+	4	4.11	5.02 ± 0.20	0.80 ± 0.00	4.22 ± 0.20 ^2^
Wet	−	8	8.23	5.25 ± 0.18	0.91 ± 0.11	4.33 ± 0.21
Dried	+	8	8.23	4.69 ± 0.22	0.80 ± 0.00	3.89 ± 0.22
Dried	−	8	8.23	5.13 ± 0.17	0.91 ± 0.11	4.22 ± 0.20
Paper						
Wet	+	8	8.23	5.80 ± 0.16	0.80 ± 0.00	5.00 ± 0.16
Wet	−	16	16.46	5.69 ± 0.18	0.30 ± 0.00	5.39 ± 0.22 ^1^
Dried	+	30	30.85	4.80 ± 0.16	1.02 ± 0.15	3.78 ± 0.22
Dried	−	16	16.46	4.91 ± 0.22	0.30 ± 0.00	4.61 ± 0.22 ^1^
Plastic						
Wet	+	16	16.46	5.91 ± 0.11	0.30 ± 0.00	5.61 ± 0.11 ^1^
Wet	−	16	16.46	5.80 ± 0.16	0.30 ± 0.00	5.50 ± 0.16 ^1^
Dried	+	30	30.85	4.58 ± 0.15	1.58 ± 0.20	3.00 ± 0.25
Dried	−	30	30.85	5.25 ± 0.18	1.47 ± 0.21	3.78 ± 0.28
**405 nm**						
Metal						
Wet	+	60	60.55	5.25 ± 0.18	0.30 ± 0.00	4.94 ± 0.18 ^1^
Wet	−	60	60.55	5.47 ± 0.17	0.30 ± 0.00	5.17 ± 0.17 ^1^
Dried	+	90	90.83	4.91 ± 0.22	1.02 ± 0.15	3.89 ± 0.27
Dried	−	60	60.55	4.47 ± 0.21	0.91 ± 0.11	3.56 ± 0.24
Paper						
Wet	+	30	30.28	4.91 ± 0.11	0.91 ± 0.11	4.00 ± 0.16
Wet	−	60	60.55	4.69 ± 0.17	0.30 ± 0.00	4.39 ± 0.17 ^1^
Dried	+	30	30.28	4.91 ± 0.18	2.58 ± 0.20	2.33 ± 0.27
Dried	−	30	30.28	4.91 ± 0.11	2.58 ± 0.22	2.33 ± 0.25
Plastic						
Wet	+	60	60.55	4.91 ± 0.11	0.80 ± 0.00	4.11 ± 0.11
Wet	−	60	60.55	4.80 ± 0.00	0.30 ± 0.00	4.50 ± 0.00 ^1^
Dried	+	90	90.83	4.47 ± 0.17	1.02 ± 0.15	3.45 ± 0.29
Dried	−	90	90.83	5.25 ± 0.20	2.99 ± 0.19	2.26 ± 0.28
**222 nm**						
Metal						
Wet	+	8	0.15	5.58 ± 0.20	0.80 ± 0.00	4.78 ± 0.20
Wet	−	16	0.31	5.69 ± 0.18	1.13 ± 0.17	4.56 ± 0.25
Dried	+	30	0.58	4.91 ± 0.18	3.25 ± 0.18	1.67 ± 0.25
Dried	−	90	1.74	5.02 ± 0.15	3.80 ± 0.24	1.22 ± 0.28
Paper						
Wet	+	16	0.31	5.58 ± 0.15	0.69 ± 0.19	4.89 ± 0.24
Wet	−	60	1.16	4.36 ± 0.18	0.30 ± 0.00	4.05 ± 0.18 ^1^
Dried	+	90	1.74	4.36 ± 0.18	1.02 ± 0.15	3.33 ± 0.23
Dried	−	60	1.16	3.69 ± 0.18	0.80 ± 0.00	2.89 ± 0.18
Plastic						
Wet	+	16	0.31	5.25 ± 0.18	0.91 ± 0.11	4.33 ± 0.21 ^2^
Wet	−	60	1.16	4.25 ± 0.18	0.30 ± 0.00	3.95 ± 0.18 ^1^
Dried	+	90	1.74	4.69 ± 0.18	1.80 ± 0.21	2.89 ± 0.28
Dried	−	90	1.74	5.80 ± 0.16	2.58 ± 0.29	3.22 ± 0.33

^1^ Log reduction was acquired by large volume culture, no infectious virus detected. ^2^ Log reduction was acquired by large volume culture.

## Data Availability

All data is contained within the article or supplementary material.

## Supplementary Materials

The following are available online at https://www.mdpi.com/article/10.3390/v13081436/s1: Figure S1, Spatial light intensities for blue LED (405 nm), germicidal UVC (254 nm), far-UVC (222 nm) and LED UVC (265 nm). All measurements were taken at 25 cm distance from the light source; Figure S2A,B, Average temperature (A) and humidity (B) measurements taken over a 90 min exposure to blue LED (405 nm), far-UVC (222 nm) and dual (405 nm + 222 nm) light in three independent experiments. Control temperature and humidity were taken at room temperature.
