# Supplementary figures and images for: Virucidal Efficacy of Blue LED and Far-UVC Light Disinfection against Feline Infectious Peritonitis Virus as a Model for SARS-CoV-2
Source: Viruses. 2021 Jul 23;13(8):1436. doi: 10.3390/v13081436 (PMC8402852; doi:10.3390/v13081436)

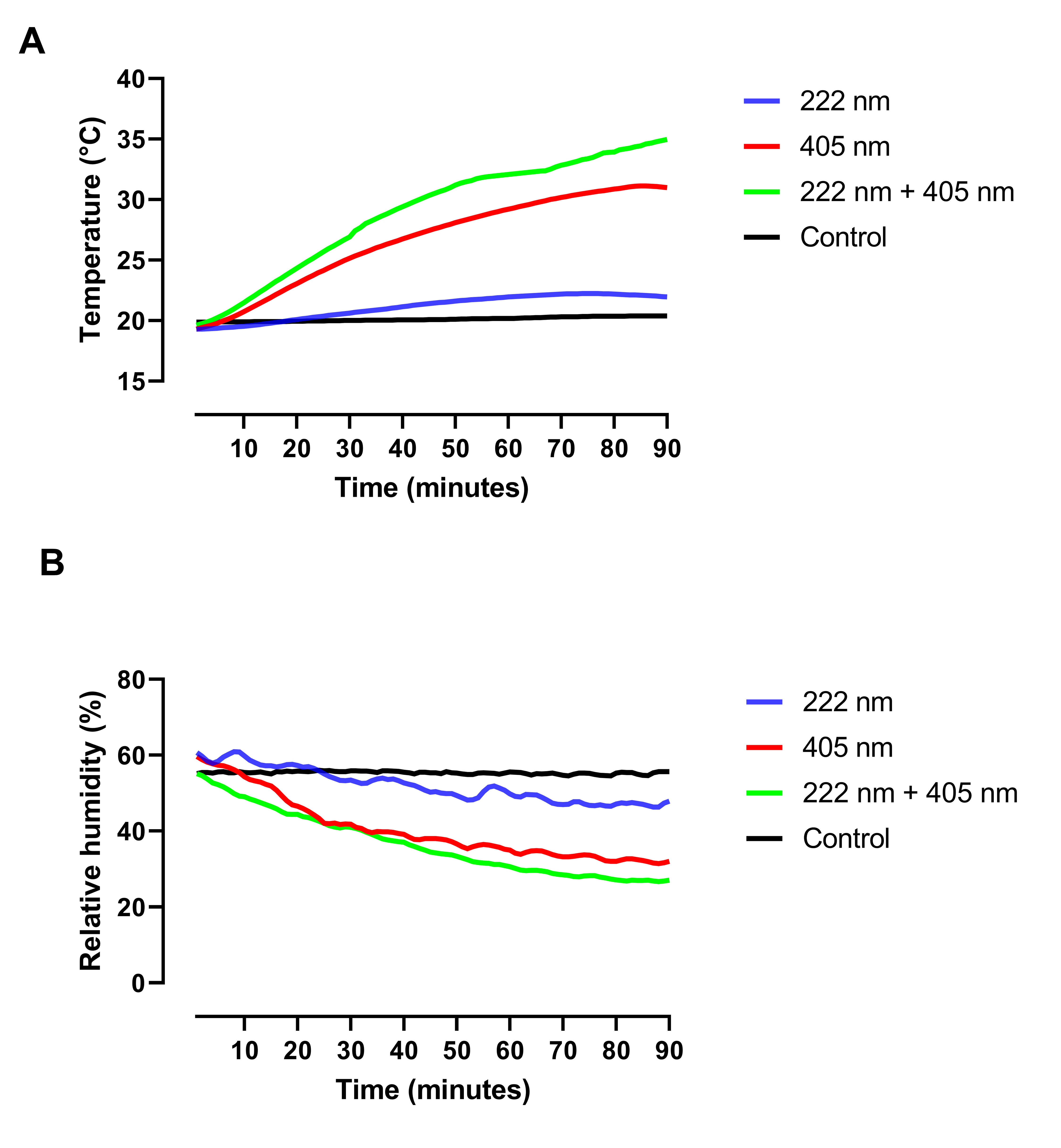

Supplement: Supplementary file 1 [file viruses-13-01436-s001.zip › Supplementary figures/Supplemenatry figure 2.tif]

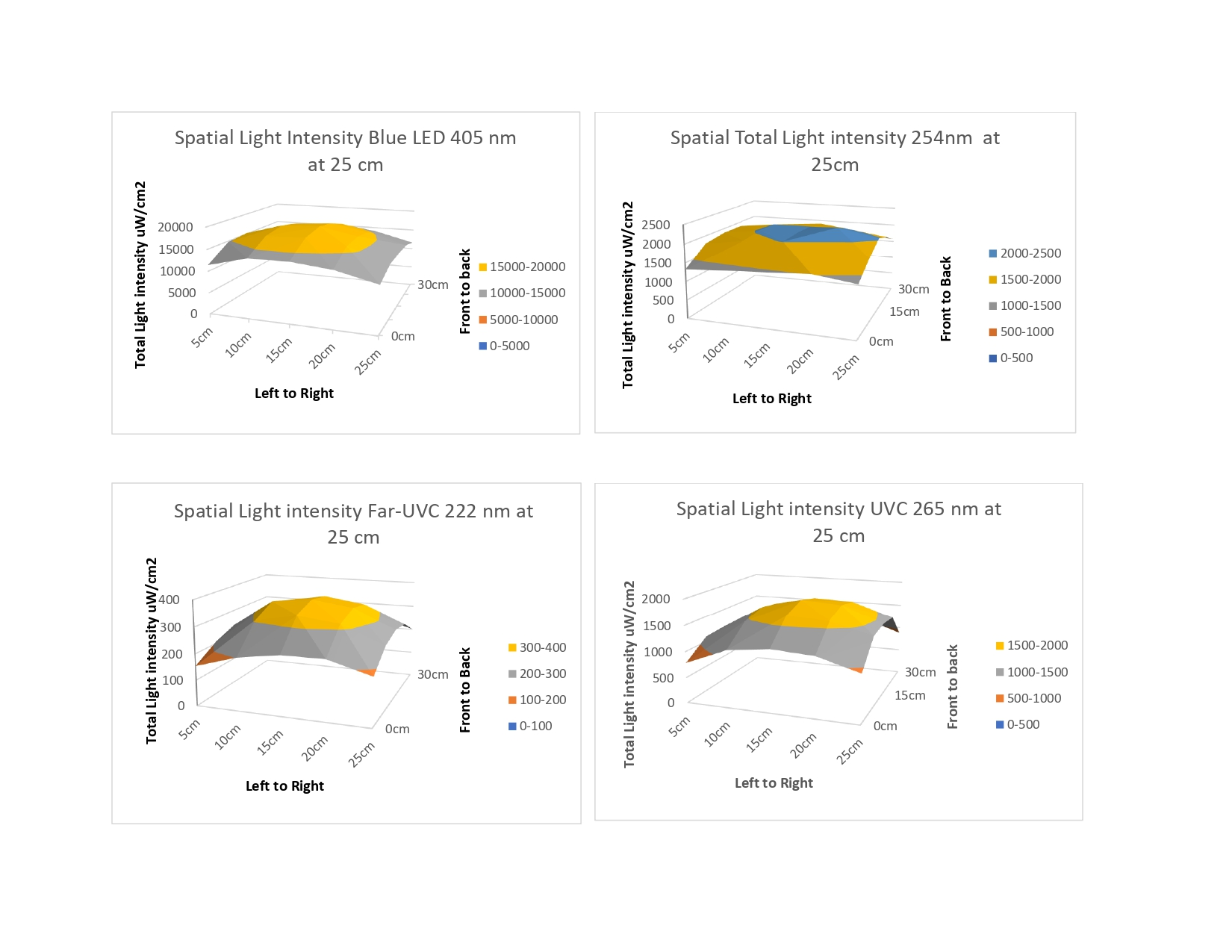

Supplement: Supplementary file 1 [file viruses-13-01436-s001.zip › Supplementary figures/Supplementary Figure 1.tif]
